# Constrained L1-Norm Minimization Method for Range-Based Source Localization under Mixed Sparse LOS/NLOS Environments

**DOI:** 10.3390/s21041321

**Published:** 2021-02-13

**Authors:** Chengwen He, Yunbin Yuan, Bingfeng Tan

**Affiliations:** 1 State Key Laboratory of Geodesy and Earth’s Dynamics, Innovation Academy for Precision Measurement Science and Technology, Chinese Academy of Sciences, Wuhan 430077, China; yybgps@apm.ac.cn (Y.Y.); bingfengtan@apm.ac.cn (B.T.); 2 University of Chinese Academy of Sciences, Beijing 100049, China

**Keywords:** line-of-sight/non-line-of-sight, source localization, constrained L1-norm minimization method

## Abstract

Under mixed sparse line-of-sight/non-line-of-sight (LOS/NLOS) conditions, how to quickly achieve high positioning accuracy is still a challenging task and a critical problem in the last dozen years. To settle this problem, we propose a constrained L1 norm minimization method which can reduce the effects of NLOS bias for improve positioning accuracy and speed up calculation via an iterative method. We can transform the TOA-based positioning problem into a sparse optimization one under mixed sparse LOS/NLOS conditions if we consider NLOS bias as outliers. Thus, a relatively good method to deal with sparse localization problem is L1 norm. Compared with some existing methods, the proposed method not only has the advantages of simple and intuitive principle, but also can neglect NLOS status and corresponding NLOS errors. Experimental results show that our algorithm performs well in terms of computational time and positioning accuracy.

## 1. Introduction

In the last few decades, localization of the mobile station (MS) is a fundamental task for lots of applications in different kinds of wireless sensor networks. One of them uses MS to receive time signals from a lot of base stations (BSs) with known coordinates, which can be transformed into distance data, e.g., time-of-arrival (TOA) measurements [1,2,3,4,5,6]. According to these measurements, the coordinates of MS can be estimated in the intersection area of different circles, whose centers and radius are the coordinates of BSs and corresponding measurements, respectively. If the intersection area becomes larger, the coordinates of MS will be difficult to be estimated and the corresponding positioning performance will be degraded. Additionally, note that Gaussian noise, NLOS error and layout of BSs are the most important factors that affect the size of the intersection area [7,8,9,10,11,12,13,14,15,16,17,18,19,20,21,22,23,24,25]. Among them, NLOS error may be the most influential factor because it can directly lead to inaccurate measurements, resulting in inaccurate localization results or even divergence [7,8,9,10,11,12,13,14,15,16,17,18,19,20,21,22,23,24,25,26,27,28,29,30,31]. Therefore, it is of great practical significance to study how to improve the positioning performance and degrade the effects of NLOS errors without requiring the priori information of NLOS status and NLOS errors. In this paper, we deal with the TOA-based localization problem by constrained L1-norm minimization technique.

To reduce the effects of NLOS errors and enhance the positioning performance, lots of methods have been widely studied in [7,8,9,10,11,12,13,14,15,16,17,18,19,20,21,22,23,24,25,26,27,28,29,30,31]. In [10], a fast identification of NLOS error using eigenvector (FINE) algorithm is proposed by using autocorrelation matrix eigenvalues of residuals to identify the NLOS paths. According to the magnitude of residuals and eigenvalues, only one NLOS path can be identified at a time. Then, corresponding BS and observation should be deleted so as to detect the next NLOS path. Only when there is no NLOS path can the positioning result be obtained with all the LOS observations. Monte Carlo simulation results show that FINE method can provide a very fast and simple way to identify the NLOS status. Nevertheless, it can also detect LOS path as NLOS path and neglect the contribution of NLOS paths, which ultimately degrades the positioning accuracy.

Furthermore, some convex optimization algorithms in [9], and in [15,16,17,18,19,20,21,22], which partially mitigate the effects of NLOS errors by using all LOS and NLOS measurements, perform well in terms of localization performance. In [9], the semi-definite relaxation (SDR) and second-order cone relaxation (SOCR) technologies are taken by using all measurements. Although they have the ability to overcome some effects of NLOS errors and achieve good results, there is still a lot of room for improvement in terms of positioning accuracy. Fortunately, based on a priori knowledge of known NLOS status, Wang et al. [9] presents another two algorithms, both of which achieve exceedingly high positioning accuracy over former methods by adding all equality constraints in LOS paths and inequality constraints in NLOS paths to convex objective function. However, two algorithms are only suitable for theoretical research and cannot be extended to the industrial market because their assumptions cannot be realized in reality. Recently, a new robust SDP algorithm (RSDP-New) is presented in [16], which performs well under mixed LOS/NLOS environments and achieves high-precision positioning by introducing a balance parameter and specifying the values of NLOS errors in advance. However, its disadvantages, which are that the constraints are too many and complicated, assumptions are unrealistic, estimated coordinates might divergence and the calculation time is too long, affect the actual application. In short, it is a very difficult task to find an algorithm that cannot only degrade the effects of NLOS errors as much as possible but also ensure better positioning accuracy and higher calculation speed without many assumptions.

In this paper, a new simple algorithm is proposed to settle above issues, which is called constrained L1-norm minimization method. Since L1-norm minimization model is convex function, no matter how far the initial coordinates are from the true position, the estimated solution can always converge to the true values by iterative method. Therefore, if the estimated coordinates are close to the real position of MS and when the number of LOS paths is more than that of NLOS paths, the localization residuals can be composed of a small number of mixed noises composed of NLOS errors and Gaussian noises and a large number of Gaussian noises. In other words, their expected values consist of a large number of Gaussian-noise variances and a small number of non-zeros which are much larger than the Gaussian-noise variances, which will cause the localization residual to be sparse vector and the localization condition to be a mixed sparse LOS/NLOS environments [32]. Even if the base station coordinates contain errors, the errors are relatively small due to the advanced measurement technology. Therefore, its influence on positioning accuracy is relatively limited, and the proposed method in this paper is still valid. Thus, the TOA-based localization problem under mixed sparse LOS/NLOS environments can be transformed into a sparsity vector problem. Based on [32], we develop a constrained L1-norm minimization method based on sparse vector technology [33,34,35,36,37,38] to settle the sparsity problem above and obtain solution quickly and efficiently, which is different from other similar methods based on low-rank matrix [35] or elastic network [20] technologies in essence. More specifically, the proposed algorithm can automatically decrease the accuracy loss caused by NLOS errors to the positioning system with no need to identify the NLOS status and estimate NLOS errors. Simulation results show superiority of our algorithm in comparison with some existing methods in terms of positioning accuracy and running time.

## 2. Problem Statement and Algorithm Development

A two-dimensional (2D) localization scenario will be considered in this section. The coordinates ${\left[x,y\right]}^{T}$ of unknown MS can be estimated by using TOA measurements, which represent the distances between MS and *N* BSs with known locations that are denoted by ${\left[x_{i},y_{i}\right]}^{T},i=1,\ldots ,N$ and contain no other errors except the Gaussian noises and NLOS errors we added. After obtaining the observations, the *i*th measurement equation or the Euclidean distance between MS and ith BS are modeled as

(1) $$\sqrt{{\left(x-x_{i}\right)}^{2}+{\left(y-y_{i}\right)}^{2}}=\left\{\begin{array}{c} d_{i}-\eta_{i},i\in \phi_{L}\\ d_{i}-\eta_{i}-\varepsilon_{i},i\in \phi_{NL} \end{array},\right.$$

where $d_{i}$ is observation value between MS and ith BS, $\eta_{i}$ is Gaussian noise, $\phi_{L}$ represents the sets of LOS paths, $\phi_{NL}$ denotes the sets of NLOS paths, and $\varepsilon_{i}$ is NLOS error which is always bigger than zero and much larger than $\left|\eta_{i}\right|$, i.e.,

(2) $$\frac{\varepsilon_{i}}{\left|\eta_{i}\right|}\gg 1.$$

Since model (1) is highly nonlinear, it needs further processing to facilitate the establishment of the new model. Hence, squaring both sides of (1) and moving $d_{i}^{2}$ to the left-hand side, we obtain

(3) $$\begin{array}{c} x^{2}+y^{2}+x_{i}^{2}+y_{i}^{2}-2x_{i}x-2y_{i}y-d_{i}^{2}\\ =\left\{\begin{array}{c} \eta_{i}^{2}-2d_{i}\eta_{i},i\in \phi_{L}\\ \eta_{i}^{2}+\varepsilon_{i}^{2}+2\varepsilon_{i}\eta_{i}-2d_{i}\left(\eta_{i}+\varepsilon_{i}\right),i\in \phi_{NL} \end{array}.\right. \end{array}$$

Taking least squares algorithm and introducing four additional variables (i.e., A, x, b and e), (3) can be equivalently written as

(4) Ax−b=e,

where

(5) $$A=\left[\begin{array}{ccc} -2x_{1} & -2y_{1} & 1\\ \vdots & \vdots & \vdots \\ -2x_{N} & -2y_{N} & 1 \end{array}\right],$$

(6) $$x=\left[\begin{array}{ccc} x & y & x^{2}+y^{2} \end{array}\right],$$

(7) $$b=\left[\begin{array}{c} d_{1}^{2}-\left(x_{1}^{2}+y_{1}^{2}\right)\\ \vdots \\ d_{N}^{2}-\left(x_{N}^{2}+y_{N}^{2}\right) \end{array}\right],$$

and residual vector $e={\left[\begin{array}{ccc} e_{1} & \cdots & e_{N} \end{array}\right]}^{T}$ is made up of most of $e_{i}=\eta_{i}^{2}-2d_{i}\eta_{i},i\in \phi_{L}$ and small part of $e_{i}=\eta_{i}^{2}+\varepsilon_{i}^{2}+2\varepsilon_{i}\eta_{i}-2d_{i}\left(\eta_{i}+\varepsilon_{i}\right),i\in \phi_{NL}$ under mixed sparse LOS/NLOS conditions. Hence, its expected values are made up of $\sigma_{i}^{2},i\in \phi_{L}$ and non-zeros (i.e., $\varepsilon_{i}^{2}+\sigma_{i}^{2}-2d_{i}\varepsilon_{i},i\in \phi_{NL}$), with most of them $\sigma_{i}^{2}$.

For each Monte Carlo simulation experiment, variable $p_{i}$ is introduced to represent the ratio relationship between NLOS error $\varepsilon_{i}$ and Gaussian noise $\eta_{i}$. Thus, we have

(8) $$p_{i}=\frac{\varepsilon_{i}}{\left|\eta_{i}\right|}\gg 1,i=1,2,\ldots ,N.$$

In order to apply the method proposed in this paper to mitigate the effects of NLOS errors as much as possible, we must guarantee that vector e has the sparse character. To prove that vector e is sparse, two prerequisites based on the definition of sparse vector in [33,34] need to be guaranteed: (1) Most of the members of vector e are $e_{i},i\in \phi_{L}$; (2) The ratio $f_{i}$ of the NLOS elements (i.e., $e_{i},i\in \phi_{NL}$) of vector e to absolute residual of LOS elements (i.e., $e_{i},i\in \phi_{L}$) needs to be much greater than 1. Based on the definition of mixed sparse LOS/NLOS conditions in Section 1, the first condition is obviously satisfied. For the second condition, following expressions will be given to derive the ratio $f_{i}$ (i.e., $\frac{e_{i},i\in \phi_{NL}}{e_{i},i\in \phi_{L}}$).

(9) $$f_{i}=\frac{\left|\eta_{i}^{2}+\varepsilon_{i}^{2}+2\varepsilon_{i}\eta_{i}-2d_{i}\left(\eta_{i}+\varepsilon_{i}\right)\right|}{\left|\eta_{i}^{2}-2d_{i}\eta_{i}\right|}.$$

Since the magnitude of $\eta_{i}$ is very small, $\eta_{i}^{2}$ can be ignored. Note that $\varepsilon_{i}$ is much larger than $\eta_{i}$ and is less than $d_{i}$. So (9) can be equivalently written as

(10) $$\begin{array}{c} f_{i}=\frac{2d_{i}\left(\left|\eta_{i}\right|+\varepsilon_{i}\right)-\varepsilon_{i}^{2}-2\varepsilon_{i}\left|\eta_{i}\right|}{2d_{i}\left|\eta_{i}\right|}\\ \;=1+\frac{\varepsilon_{i}}{\left|\eta_{i}\right|}-\frac{\varepsilon_{i}}{2\left|\eta_{i}\right|}\cdot \frac{\varepsilon_{i}}{d_{i}}-\frac{\varepsilon_{i}}{d_{i}}\\ \;≻p_{i}\left(1-\frac{1}{2}\cdot \frac{\varepsilon_{i}}{d_{i}}\right). \end{array}$$

Based on (10), two cases need to be analyzed. If $\varepsilon_{i}\ll d_{i}$, $f_{i}≻p_{i}\gg 1$ can be obtained and the second condition is strongly satisfied. If $\varepsilon_{i}\approx d_{i}$ and $\varepsilon_{i}≺d_{i}$, $f_{i}≻\frac{1}{2}p_{i}≻1$ can be acquired and the second condition can be considered to be satisfied to some extent just for $p_{i}\gg 1$. Therefore, we can infer that vector e is sparse and the proposed algorithm can be used in sparse optimization problem.

Combined with the above reasonable analysis, a good way to solve the TOA-based localization problem under mixed sparse LOS/NLOS conditions is constraint L1-norm, which is the difference with the model of [32] and can be modeled as

(11) $$\begin{array}{c} \min_{x}\;{∥Ax-b∥}_{1}\\ s.t.\;x^{T}Px+q^{T}x=0, \end{array}$$

where $P=\mathrm{diag}\left(\left[1,1,0\right]\right)$, $q={\left[0,0,-1\right]}^{T}$, and ${∥\cdot ∥}_{1}$ stands for L1-norm minimization criterion defined as ${∥z∥}_{1}=\left(\sum_{i}{\left|z_{i}\right|}^{1}\right)$. By introducing a new vector z, (9) can be equivalently rewritten as

(12) $$\begin{array}{c} \min_{x}\;{∥z∥}_{1}\\ s.t.\;z=\mathrm{Ax}-b,\\ \;x^{T}\mathrm{Px}+q^{T}x=0. \end{array}$$

Using augmented Lagrangian, (12) can be further represented as

(13) $$\begin{array}{c} \min_{x}\;{∥z∥}_{1}-w^{T}\left(\mathrm{Ax}-b-z\right)+\frac{\rho }{2}{∥\mathrm{Ax}-b-z∥}_{2}^{2}\\ s.t.\;x^{T}\mathrm{Px}+q^{T}x=0, \end{array}$$

which can be simplified as

(14) $$\begin{array}{c} \min_{x}\;{∥z∥}_{1}+\frac{\rho }{2}{∥\mathrm{Ax}-b-z-\frac{w}{\rho }∥}_{2}^{2}\\ s.t.\;x^{T}\mathrm{Px}+q^{T}x=0. \end{array}$$

To settle problem (14) quickly and effectively, we divide the solution process into two steps. Firstly, taking alternating direction method of multipliers (ADMM) [35,36,37] to deal with the object function of (14) and ignoring the constraint, vector **z**, **w** and **x** can be obtained.

(15) zk+1=S11ρρg.

(16) $$w^{k+1}=w^{k}-\rho \left(Ax-b-z\right).$$

(17) $$x^{k+1}={\left(A^{T}A\right)}^{-1}A^{T}\left(b+r+\frac{w}{\rho }\right).$$

where

(18) $$g=Ax-b-r-\frac{w}{\rho },$$

and w is lagrange multiplier, ρ is penalty parameter, S11ρρg is an operator, whose specific calculation process is detailed in [36,37,38] and can be written as

(19) S11ρρgi=signgi·maxgi−1ρ,0,

where

(20) $$sign\left(g_{i}\right)=\left\{\begin{array}{c} 1,g_{i}>0\\ 0,g_{i}=0\\ -1,g_{i}<0 \end{array}.\right.$$

Next, considering the constraint of (14) and using Lagrangian, (14) can be denoted as

(21) $$\begin{array}{c} L\left(z,x,w,v\right)=\\ {∥z∥}_{1}+\frac{\rho }{2}{∥Ax-b-z-\frac{w}{\rho }∥}_{2}^{2}+v\left(x^{T}\mathrm{Px}+q^{T}x\right). \end{array}$$

To get the iterative solution *v*, we introduce a new vector **m** and set the derivative of Formula (18) with respect to x equal 0. Thus, we have

(22) $$\frac{\partial L}{\partial x}=\rho A^{T}\left(Ax-b-z-\frac{w}{\rho }\right)+2v\mathrm{Px}+vq=0.$$

(23) m=−ρATAx−b−z−wρ−ρATAx−b−z−wρ2Px+q2Px+q.

(24) v=∑i=13mi∑i=13li33.

By substituting (24) into (21), the positioning solution of the current cycle is

(25) $${\tilde{x}}^{k+1}={\left(H^{T}H\right)}^{-1}H^{T}\left(\rho A^{T}\left(b+z+\frac{w}{\rho }\right)-vq\right).$$

where $H=\rho A^{T}A+2vP.$

The approximate calculation process of our method can be summarized as follows:

(1) Firstly, we set $w^{0}=1_{N\times 1}$, $\rho^{0}=0.01$ and $x^{0}={\left[1,1,2\right]}^{T}$ as the initial iteration value of method ADMM. It should be noted that since the constrained L1-norm model is convex function, any value of $x^{0}$ can be used to obtain the convergence solution via ADMM algorithm.
(2) Then, recycle computing Formula (13) to (20) until both Ax−b and $x^{T}\mathrm{Px}+q^{T}x$ are smaller than the threshold, and output the final positioning solution under the current Monte Carlo simulation experiment.

Special attention should be paid to consult [36] to obtain the adaptive iteration process of ρ for speeding up the convergence speed of algorithm.

## 3. Simulation Results

**Figure 1:**
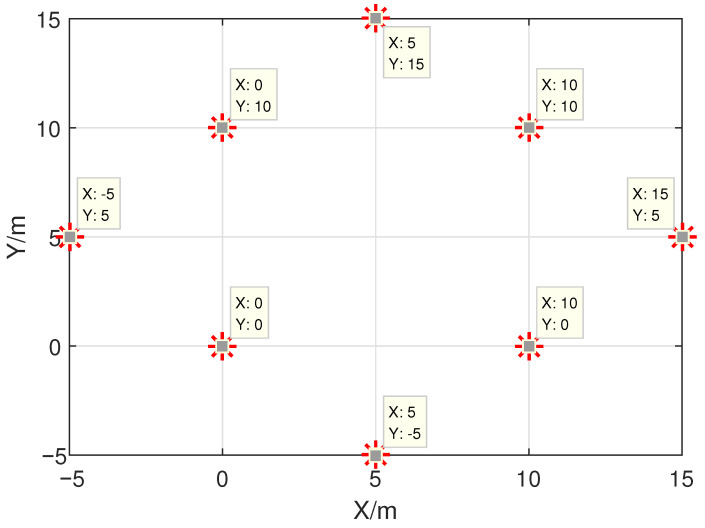
Coordinate diagram of base stations.

This section gives six examples to test the positioning accuracy and calculation speed of the proposed algorithm (i.e., CL1). In order to highlight the advantages of our algorithm, we consider SDP [9], SOCR [9] and constrained weighted least squares (CWLS) [2] algorithms as comparison algorithms for research. Additionally, the localization system is made up of eight sensors, which are located at $\left(0,0\right)$, $\left(10,0\right)$, $\left(10,10\right)$, $\left(0,10\right)$, $\left(15,5\right)$, $\left(5,-5\right)$, $\left(5,15\right)$ and $\left(-5,5\right)$, and their layout is show in Figure 1. Assuming that Gaussian noise $\eta_{i}$ with mean zero and variance $\sigma_{i}^{2}$ and NLOS error $\varepsilon_{i}$ with with randomly generated between $6*\max\left(\left|\eta_{i}\right|\right),i=1,2,\ldots ,N$ and $12*\max\left(\left|\eta_{i}\right|\right),i=1,2,\ldots ,N$ are the basic conditions for the following six experiments. Furthermore, root mean square error (RMSE), denoted by $\mathrm{RMSE}=\sqrt{\frac{1}{M}\sum_{i=1}^{M}{\left(\tilde{x_{i}}-\bar{x_{i}}\right)}^{2}}$, with $\tilde{x_{i}}$ and $\bar{x_{i}}$ being the estimated position and true position of MS, can be used as an indicator to evaluate the precision performance and the number of simulation times is M=300 for each noise variance or BSs layout. In all experiments, coordinates of MS are randomly generated within the area surrounded by BSs.

**Figure 2:**
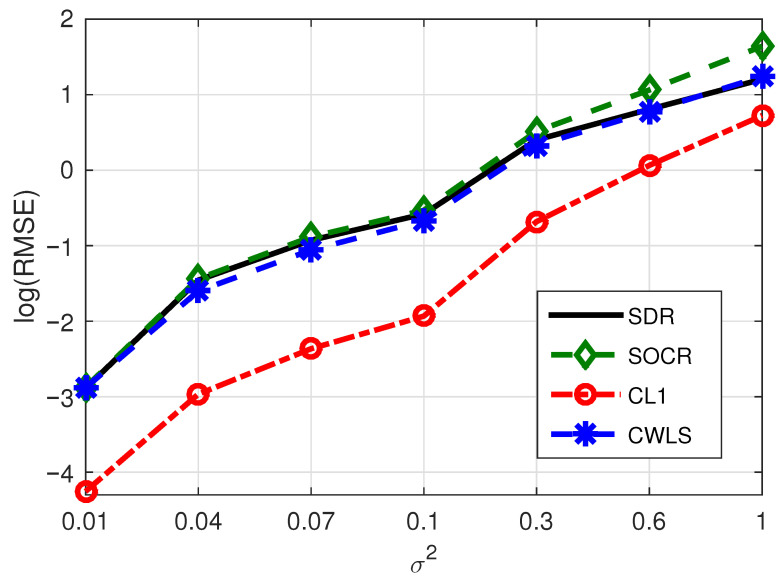
RMSE comparison of four methods under the conditions of six BSs and one NLOS.

**Example** **1.** *In this example, we randomly select six BSs from eight BSs and fix the number of NLOS paths as one to test the localization accuracy of different methods. Corresponding positioning performance of four algorithms are shown in Figure 2, from which we see that the CL1 method is significantly superior to SDR, CWLS and SOCR methods, indicating that L1-norm can also be applied to the localization problem in a simple and direct way. Since L2 norm is mainly applicable to Gaussian white noise environment and L1 norm is applicable to sparse environment, it is normal that SDR, SOCR and CWLS methods, which based on L2 norm, have poor performance.*

**Figure 3:**
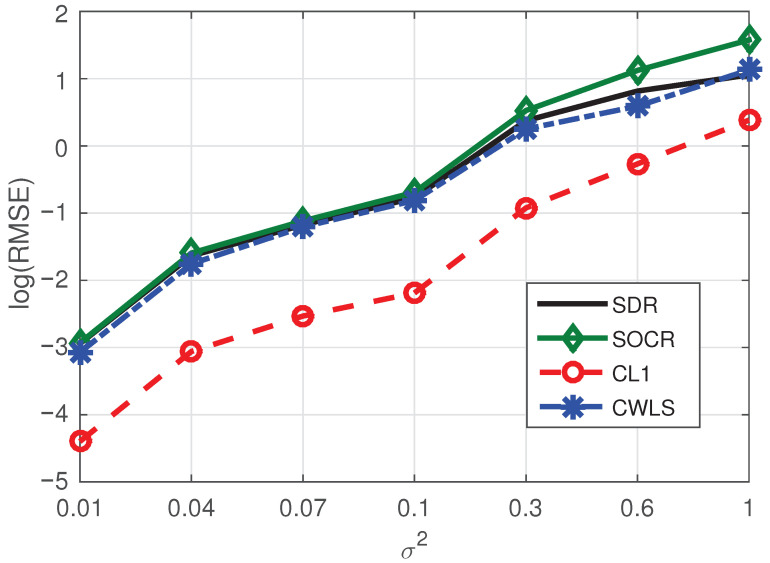
RMSE comparison of four methods under the conditions of seven BSs and one NLOS.

**Example** **2.** *In this example, we set the number of NLOS paths and BSs as one and seven, respectively. Corresponding simulation results are shown in Figure 3. Obviously, CL1 method still performs better than the other three algorithms. Since sparse method has great advantages in sparse environment, it is normal that our method based on sparse technology has good advantages.*

**Figure 4:**
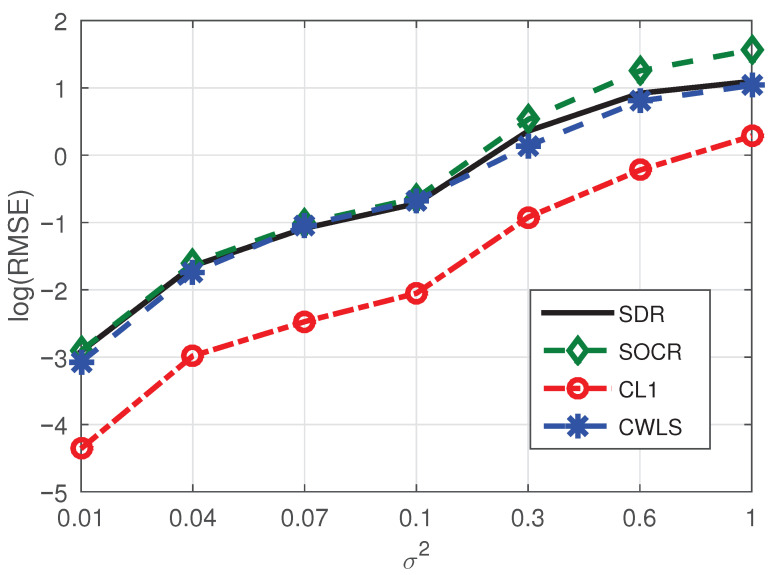
RMSE comparison of four methods under the conditions of seven BSs with noises and one NLOS.

**Example** **3.** *To test the positioning performance of the proposed method when BSs contain errors, we add Gaussian white noise with mean 0 and variance 0.02 to the BSs based on Example 2. Corresponding localization results are shoun in Figure 4. Obviously, both Figure 3 and Figure 4 are basically consistent in terms of curve values and trends, indicating that BSs with noise have little influence on our method.*

**Figure 5:**
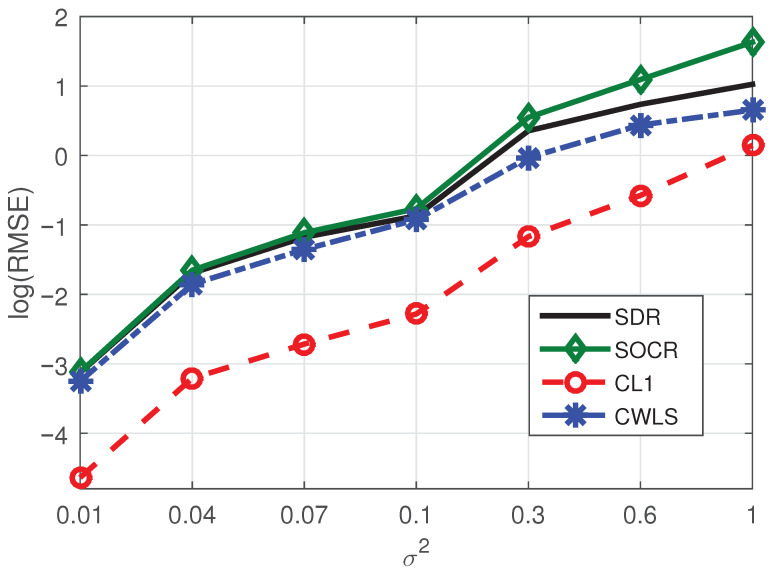
RMSE comparison of four methods under the conditions of eight BSs and one NLOS.

**Example** **4.** *In this test, we set the number of BSs as eight and fix the number of NLOS paths as one to further test the performance of CL1 method. Corresponding simulation results are shown in Figure 5 and corresponding conclusions are consistent with Example 1, Example 2 and Example 3.*

**Figure 6:**
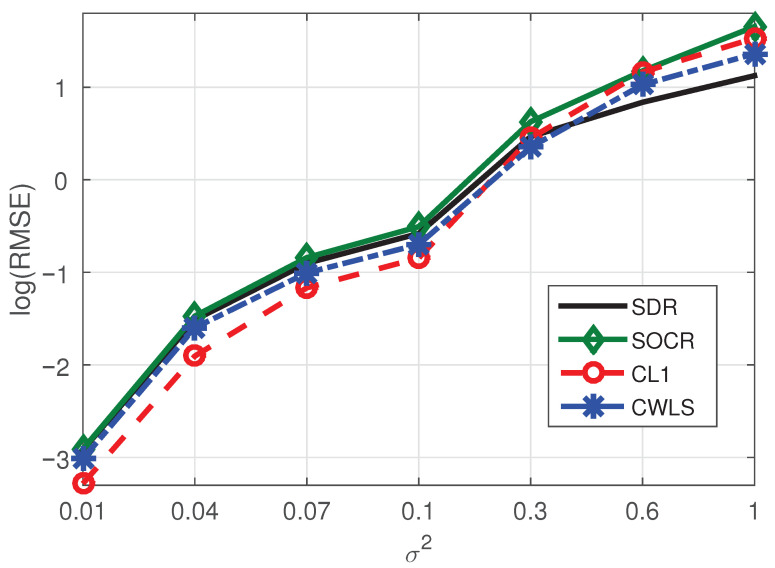
RMSE comparison of four methods under the conditions of eight BSs and two NLOS.

**Example** **5.** *To further verify the localization performance of the CL1 algorithm, we set the number of NLOS paths as two and other conditions are consistent with Example 4. The simulation results are shown in Figure 6, from which we see that the performance of CL1 method is superior to SDR, CWLS and SOCR methods in low-noise scenario, and the performance of four algorithms is almost the same in the high-noise condiiton.*

**Example** **6.** *To test the calculation speed of the four algorithms above, we characterize it by calculating the time consumed by each algorithm in the process of running each program 400 times. Based on the conditions of example 3, we set $\sigma_{i}^{2}=0.1$ and then the result of each computation time will be shown in the Table 1. We see that the algorithm with the shortest time is CL1 method, followed CWLS and SOCR methods, and finally SDR method. There will be some differences between above four algorithms running on different computers, different software, and different environments, but the ratio of computational time between algorithms will not change. Additionally, our algorithm needs to iterate 70 times to reach the convergence solution on average.*

Consequently, considering the experimental test results shown in Figure 2, Figure 3, Figure 4, Figure 5 and Figure 6 and Table 1, we can draw the following conclusions:

(1) The proposed algorithm has great advantages in positioning accuracy and has some advantages in computational speed.
(2) Even if the base station contains noise, its influence on the positioning performance of the proposed algorithm is limited.
(3) When the number of BSs increases while the number of NLOS paths remains constant, the positioning performance of the proposed method will be improved due to the increase of sparsity. On the contrary, the BSs remains unchanged, and when the number of NLOS paths increases, the localization performance of our method will be reduced due to the decrease of sparsity.

Although our algorithm is currently only applicable to the mixed sparse LOS/NLOS environments, it is still of reference value to the academic community. In the future, we will devote ourselves to the study of high-precision positioning algorithms in non-sparse environment or NLOS condition.

## 4. Conclusions

In this paper, we propose a constrained L1-norm minimization method, simple and efficient, to improve the positioning accuracy and speed up calculation under mixed sparse LOS/NLOS conditions. Based on the conditions that the magnitudes of NLOS errors are much larger than the Gaussian noises and the number of LOS paths is larger than that of NLOS paths, the residuals can form a sparse vector which fully guarantees that our algorithm is innovative and feasible in principle. Meanwhile, experimental simulation results also confirm that the proposed algorithm not only has the advantage of fast computation speed, but also can achieve a high positioning accuracy with no need to identify NLOS status and estimate NLOS errors.

## Figures and Tables

**Table 1:** Average computation time (in seconds) of four algorithms.

Method	Average Time
SDR	1.43
SOCR	0.91
CL1	0.009
CWLS	0.53

## Data Availability

Not applicable.
